# Inflammation-driven bone formation in a mouse model of ankylosing spondylitis: sequential not parallel processes

**DOI:** 10.1186/s13075-015-0805-0

**Published:** 2016-01-29

**Authors:** Hsu-Wen Tseng, Miranda E. Pitt, Tibor T. Glant, Allan F. McRae, Tony J. Kenna, Matthew A. Brown, Allison R. Pettit, Gethin P. Thomas

**Affiliations:** The University of Queensland Diamantina Institute, Translational Research Institute, 37 Kent Street, Woolloongabba, QLD 4102 Australia; Section of Molecular Medicine, Department of Orthopedic Surgery, Rush University Medical Center, 1735 W. Harrison Street, Cohn Research Building, Chicago, IL 60612 USA; The University of Queensland, Queensland Brain Institute, QBI Building, St Lucia, QLD 4072 Australia; The University of Queensland-Mater Research Institute, Translational Research Institute, 37 Kent Street, Woolloongabba, QLD 4102 Australia

**Keywords:** Ankylosing spondylitis, Proteoglycan-induced spondylitis mouse model, Enthesis, Intervertebral disc destruction, Chondroidal bone formation, Spondyloarthropathy, Arthritis, Osteoproliferation

## Abstract

**Background:**

Ankylosing spondylitis (AS) is an immune-mediated arthritis particularly targeting the spine and pelvis and is characterised by inflammation, osteoproliferation and frequently ankylosis. Current treatments that predominately target inflammatory pathways have disappointing efficacy in slowing disease progression. Thus, a better understanding of the causal association and pathological progression from inflammation to bone formation, particularly whether inflammation directly initiates osteoproliferation, is required.

**Methods:**

The proteoglycan-induced spondylitis (PGISp) mouse model of AS was used to histopathologically map the progressive axial disease events, assess molecular changes during disease progression and define disease progression using unbiased clustering of semi-quantitative histology. PGISp mice were followed over a 24-week time course. Spinal disease was assessed using a novel semi-quantitative histological scoring system that independently evaluated the breadth of pathological features associated with PGISp axial disease, including inflammation, joint destruction and excessive tissue formation (osteoproliferation). Matrix components were identified using immunohistochemistry.

**Results:**

Disease initiated with inflammation at the periphery of the intervertebral disc (IVD) adjacent to the longitudinal ligament, reminiscent of enthesitis, and was associated with upregulated tumor necrosis factor and metalloproteinases. After a lag phase, established inflammation was temporospatially associated with destruction of IVDs, cartilage and bone. At later time points, advanced disease was characterised by substantially reduced inflammation, excessive tissue formation and ectopic chondrocyte expansion. These distinct features differentiated affected mice into early, intermediate and advanced disease stages. Excessive tissue formation was observed in vertebral joints only if the IVD was destroyed as a consequence of the early inflammation. Ectopic excessive tissue was predominantly chondroidal with chondrocyte-like cells embedded within collagen type II- and X-rich matrix. This corresponded with upregulation of mRNA for cartilage markers *Col2a1*, *sox9* and *Comp*. Osteophytes, though infrequent, were more prevalent in later disease.

**Conclusions:**

The inflammation-driven IVD destruction was shown to be a prerequisite for axial disease progression to osteoproliferation in the PGISp mouse. Osteoproliferation led to vertebral body deformity and fusion but was never seen concurrent with persistent inflammation, suggesting a sequential process. The findings support that early intervention with anti-inflammatory therapies will be needed to limit destructive processes and consequently prevent progression of AS.

**Electronic supplementary material:**

The online version of this article (doi:10.1186/s13075-015-0805-0) contains supplementary material, which is available to authorized users.

## Background

Ankylosing spondylitis (AS) is a chronic inflammatory arthritis predominantly affecting the axial skeleton, particularly the spine and pelvis. The disease is characterised by inflammation at the entheses, which is followed by uncontrolled osteoproliferation that often leads to fusion (ankylosis) of affected joints. There is ongoing debate regarding whether inflammation is the direct trigger of the osteoproliferation seen in AS. Whilst there is evidence that the anti-inflammatory treatments of non-steroidal anti-inflammatory drugs (NSAIDs) and tumour necrosis factor (TNF)-inhibitor biologic therapies can slow osteoproliferative disease, the outcome is variable and the effect is at best partial with ankylosis still progressing. Ankylosis in patients with AS results from the development and fusion of syndesmophytes, defined as bony bridges growing at the vertebral corners. Multiple mechanisms of bone formation have been proposed to underlie the osteoproliferation in AS, including intramembranous, endochondral and chondroidal ossification [1, 2]. The causative pathological mechanism that initiate and perpetuate the excessive bone formation and subsequently ankylosis remain unknown.

In patients with AS, longitudinal magnetic resonance imaging (MRI) and radiographic imaging can be used to follow disease progression, but the resolution and sensitivity of these imaging modalities are insufficient to show correlations of specific pathological processes at a detailed anatomical level. Collection of serial biopsies from spinal lesions of patients with AS would substantially aid elucidation of mechanistic changes underlying the inflammatory response and the mechanism(s) of bone formation. However, this approach is constrained because of ethical limitations and biopsy collection challenges. A few studies have been performed on sacroiliac joint biopsies from patients undergoing hip replacement and in zygapophyseal joints from spinal surgeries [2–6]. Such studies, though informative, are limited by sample size and anatomical location, coupled with being predominantly obtained from late-stage disease patients in whom the inflammation-osteoproliferative transition has already occurred. Disease-relevant animal models thus present the only viable approach to elucidate mechanisms underlying disease progression in the axial skeleton. Time course studies in spinal samples from animal models present a powerful approach to biologically map the full progression of AS, particularly to investigate the mechanistic link between inflammation and osteoproliferation.

We aimed to delineate the disease progression and causal association between inflammation, destruction and osteoproliferation in AS by undertaking a comprehensive time course study in the proteoglycan (PG)-induced spondylitis (PGISp) mouse model. The PGISp mouse model was chosen for its recapitulation of AS clinical features, including development of both inflammation and osteoproliferation in the spine in response to an immune stimulus [7, 8]. These previous studies in the PGISp model demonstrated intervertebral disc (IVD) destabilization, cartilage damage, chondrophyte/osteophyte formation and their subsequent fusion. However, these studies were qualitative and the inter-dependence between these features was not assessed.

We performed a 6-month time course in PGISp mice spanning from the onset to advanced disease with regular sampling occurring throughout disease progression. We developed a novel histological scoring system that measured the full breadth of disease features. Unbiased computational modelling of these scores allowed us to determine the key features differentiating different disease stages and predict associations. We used histology, immunohistochemistry, and gene expression data to precisely characterise the timing and morphology of pathological events within affected joints.

## Methods

### Mouse model, immunization and collection

The PGISp model was established by treating 3-month-old female IL-4^−/−^ BALB/c mice with 2 mg of human cartilage extract (PG) together with 2 mg of dimethyl dioctadecyl ammoniumbromide (DDA) (Sigma-Aldrich, St. Louis, MO, USA) as described previously [8]. IL-4^−/−^mice were used because of the higher disease incidence and severity over wild-type BALB/c mice [9]. PG was administered via intraperitoneal injections at week 0, 3 and 6 of the study. Age-matched naïve female IL-4^−/−^ BALB/c mice were used as controls.

Spine samples, including thoracic and lumbar spine, from 12 to 17 mice were collected 6, 8, 10, 12, 16 and 24 weeks after the first PG injection. All experiments were approved by the University of Queensland animal ethics committee.

### Histology

Spine samples from 5 to 10 mice per group were fixed in 4 % paraformaldehyde (Sigma-Aldrich) for 48–72 hours followed by decalcification in 14 % ethylenediaminetetraacetic acid disodium salt dihydrate (EDTA) (Sigma-Aldrich) for 3–4 weeks. Embedded spine samples were cut into 5-μm serial sections along the sagittal plane using a standard rotary microtome. Sections were deparaffinised with xylene and graded ethanol, rehydrated in tris-buffered saline (TBS) (50 mM Tris, 150 mM NaCl, pH = 7.4), stained with Mayer’s haematoxylin and eosin (H&E) or toluidine blue (0.01 % toluidine blue O (Sigma-Aldrich) in 0.1 % sodium chloride solution, pH = 2.0) and mounted.

#### Semi-quantitative histological scoring

Histological scoring criteria outlined in Table 1 were developed to capture the broad range of inflammatory, catabolic and anabolic changes that occur within the vertebral joints of the spine in this AS model. Scoring was performed on one H&E and one toluidine blue stained section from each sample. Only samples with correct sagittal orientation and at least six IVDs in the section plane were included in the analysis. On average, nine vertebral joints were scored in each mouse. Anterior and posterior sides of each joint were scored separately and then averaged to generate the final score for any given joint. Joints with a score above zero within any of the criteria categories were defined as affected joints, and mice that had at least one affected IVD were defined as affected mice. PGISp mice with normal spine morphology and no evidence of disease were excluded from further spinal disease incidence and progression analysis. The disease progression was measured by averaging scores of all vertebral joints.

**Table 1:** Histological score criteria

Category	Score	Criteria
Inflammation	0	Normal
	1	Minor infiltration of inflammatory cells at periphery of the joint
	2	Moderate infiltration – inflammatory pannus < 50 % joint area
	3	Marked infiltration – inflammatory pannus > 50 % joint area
Intervertebral disc destruction	0	Normal
	1	Less than 50 % disc destruction
	2	More than 50 % disc destruction
	3	Total disc destruction/only necrotic disc left
Cartilage damage	0	Normal
	1	Some loss of articular cartilage or growth plate cartilage or both
	2	Severe loss of articular cartilage and some growth plate cartilage damage
	3	Severe loss of articular cartilage and severe growth plate cartilage damage
Bone erosion	0	Normal
	1	One or a few small areas of resorption in original vertebral bone.
	2	Numerous areas of obvious focal resorption in original vertebral bone or several areas of severe destruction.
Excess tissue formation (excluding inflammatory infiltrate)	0	Normal
	1	Mesenchymal cell invasion or expansion or both
	2	Moderate fibrocartilage formation (<50 % of the original disc area)
	3	Extensive fibrocartilage formation (>50 % of the original disc area)
Ectopic chondrocytes/chondrophyte	0	Normal
	1	Single small area
	2	Single large area
	3	Multiple areas

### Immunohistochemistry

Sagittal sections (4-μm) were rehydrated and subjected to different antigen retrieval processes depending on the primary antibody. For type I and II collagen, spine sections were digested in pre-warmed 25 % trypsin (Biocare Medical, Concord, NSW, Australia) for 10 min at room temperature (RT). For type X collagen, sections were incubated in 0.1 U/ml chondroitinase ABC (Sigma-Aldrich) in tris-acetate buffer (0.1 M Tris, 30 mM Acetate buffer, 10 mM EDTA, pH 6.5) for 2 hours at 37 °C. No antigen retrieval was performed for osterix antibody. All sections were incubated in 3 % H_2_O_2_ for 30 min at RT and blocked in Sniper Blocking Reagent (Biocare Medical) for 10 min followed by 10 % foetal bovine serum for 1 hour at RT. For type I or II collagen and osterix staining, sections were incubated with rabbit anti-type I or II collagen or osterix antibodies or equivalent concentration of normal rabbit IgG for 1 hour at RT. For type X collagen staining, sections were incubated with rabbit anti-type X collagen anti-serum or equivalent concentration of rabbit serum overnight at 4 °C. After washing in TBS, sections were incubated in MACH1 HRP polymer reagent (Biocare Medical) for 30 min and detected by using diaminobenzidine (DAB) chromogen (Biocare Medical). Sections were counterstained in acidified haematoxylin (Sigma-Aldrich). Reagent details are listed in Additional file 1: Table S1.

### RNA extraction and quantitative real-time polymerase chain reaction

Spine samples from five to eight PGISp or from five to seven age-matched naïve female mice at each time point were flash-frozen in liquid nitrogen and stored at −80 °C. Spine samples were homogenized separately in Trizol (Life Technologies, Mulgrave, Victoria, Australia), and RNA extracted as described previously [8]. Synthesis of cDNA was conducted by using a Tetro cDNA synthesis kit (Bioline, Alexandria, NSW, Australia) in accordance with the instructions of the manufacturer. Gene expression was measured by SYBR Green-based quantitative real-time reverse transcription polymerase chain reaction (qPCR) using the Sensimix SYBR kit and run on a ViiA7 Real-Time PCR System (Life Technologies). Primer sequences are listed in Additional file 1: Table S2. PCR conditions were 10 min at 95 °C followed by 50 cycles of 15 sec at 95 °C and 45 sec at 60 °C. The expression of individual genes was normalized to β-actin and gene expression quantified by using the ΔCT equation.

### Statistics

Data was analysed by Mann-Whitney or Kruskal-Wallis tests analysis by using PRISM 6 (GraphPad Software, La Jolla, CA, USA). *P* values of less than 0.05 were considered significant. Unsupervised analysis was conducted by using the hclust function in R. Histological scores of affected IVD of affected mice were averaged and computed by using hierarchical cluster analysis with Ward’s method.

## Results

### PGISp axial joint histopathology progresses from inflammation to osteoproliferation

To characterise axial disease progression in PGISp mice, H&E stained sections from spines collected across a 24-week disease time course were semi-quantitatively scored in accordance with the criteria outlined in Table 1. These criteria scored inflammation, vertebral disc destruction, bone erosion, excess tissue formation (mesenchymal tissue expansion or fibrocartilage formation) and ectopic chondrocyte/chondrophyte formation in the vertebral joints. Histological scoring of these major disease features was used to model axial disease progression in the PGISp mice. All the vertebral joints and IVD in naïve mice appeared normal throughout the time course and were treated as a single mouse group for statistical analysis.

**Fig. 1:**
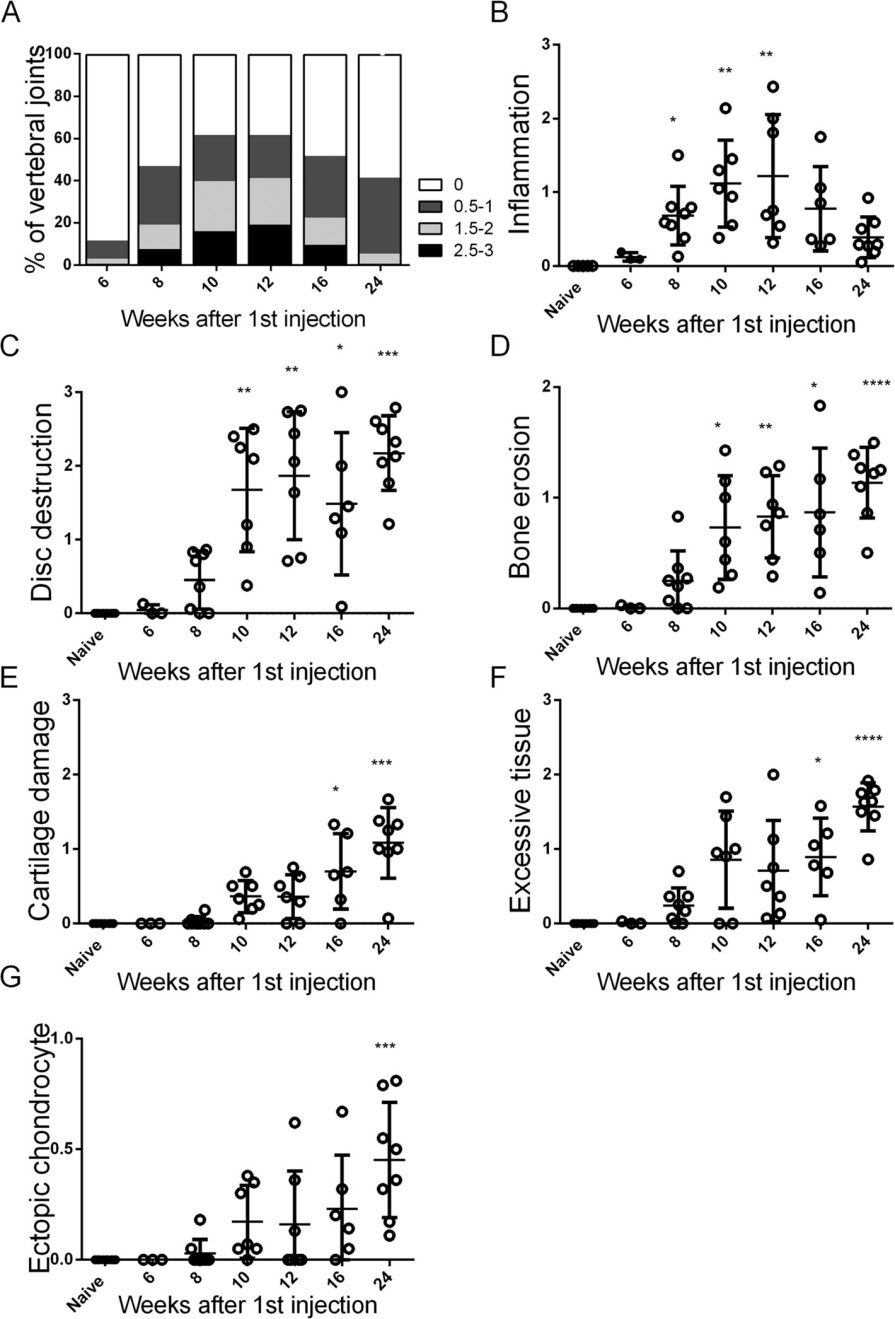
PGISp mouse model disease progression. **a** The severity of inflammatory infiltrate is represented by the percentages of different scores at each time point. Axial disease progression is described by the features of (**b**) inflammation, (**c**) disc destruction, (**d**) bone erosion, (**e**) cartilage damage, (**f**) excessive tissue formation and (**g**) ectopic chondrocyte formation. Each point represents the average scores of all vertebral joints within each affected mouse at the particular time points (3–10 mice per time point). The results are shown as mean ± standard deviation and analysed by Kruskal-Wallis test followed by Dunn’s multiple comparison test. ****P < 0.0001, ***P < 0.001, **P < 0.01, *P < 0.05 compared with naïve

The PGISp mice exhibited early disease by 6 weeks post-PG priming, and 60 % of mice had either one or two mildly affected vertebral joints. By 8 weeks after the first PG injection, every PGISp mouse had at least one affected vertebra, and 50 % of all vertebral joints were affected (Fig. 1a). The majority of joints were only mildly affected at 8 weeks, but severe inflammation was observed in a small percentage (Fig. 1a). Averaging the inflammatory scores of all the vertebral joints in individual affected PGISp mice and comparing this average inflammation score across the time course showed that inflammation peaked at 12 weeks (Fig. 1b), and 18–19 % of affected joints were severely inflamed (Fig. 1a). At both 10 and 12 weeks after PG priming, the inflammation score was significantly elevated (Fig. 1b). There was heterogeneity in inflammation severity at any time point both between and within individual animals, and some vertebral joints showed no inflammation whereas others had significant inflammatory infiltrates (Fig. 1b). Average inflammation severity decreased from 16 weeks after PG priming, and by 24 weeks the majority of joints had no or only very minor associated inflammation (Fig. 1a). When compared with all other histopathological features scored (Fig. 1c–g), inflammation was the earliest detectable axial histopathological feature in the PGISp model but was a transient disease process (Fig. 1b).

Histopathological features of the disease reflecting destruction (Fig. 1c–e) or tissue formation (Fig. 1f and g) demonstrated a sustained or progressive disease pattern. Most reached maximal score at 24 weeks after priming (Fig. 1c–e), much later than was observed for inflammation scores (Fig. 1b). Initiation of disc destruction (Fig. 1c), bone erosion (Fig. 1d), cartilage damage (Fig. 1e) and excessive tissue formation (Fig. 1f) were evident from 8 weeks after priming. Disc destruction and bone erosion were significantly increased from week 10 onwards in PGISp compared with naïve mice (Fig. 1c and d, respectively). Disc destruction (Fig. 1c) and bone erosion (Fig. 1d) peaked at the same time as inflammation (Fig. 1b) but subsequently plateaued, consistent with their being irreparable tissue changes driven by the inflammatory processes. Excessive tissue (Fig. 1e) and ectopic chondrocyte formation (Fig. 1f) increased steadily from week 10 through to week 24.

A notable feature was that, in all the mice analysed, severe inflammation and significant excessive tissue formation did not occur simultaneously within the same joint, consistent with these features representing distinct early and late stages of PGISp progression, respectively. Another feature of late-stage disease was cartilage damage, which was not significantly elevated compared with naïve or 6-week mice until 16 weeks after PG priming. This suggests that this destructive event is more likely a secondary consequence of other joint changes initiated by the inflammatory process, rather than a direct consequence of the inflammation.

### Inflammation drives tissue damage and disc destruction in PGISp mice

**Fig. 2:**
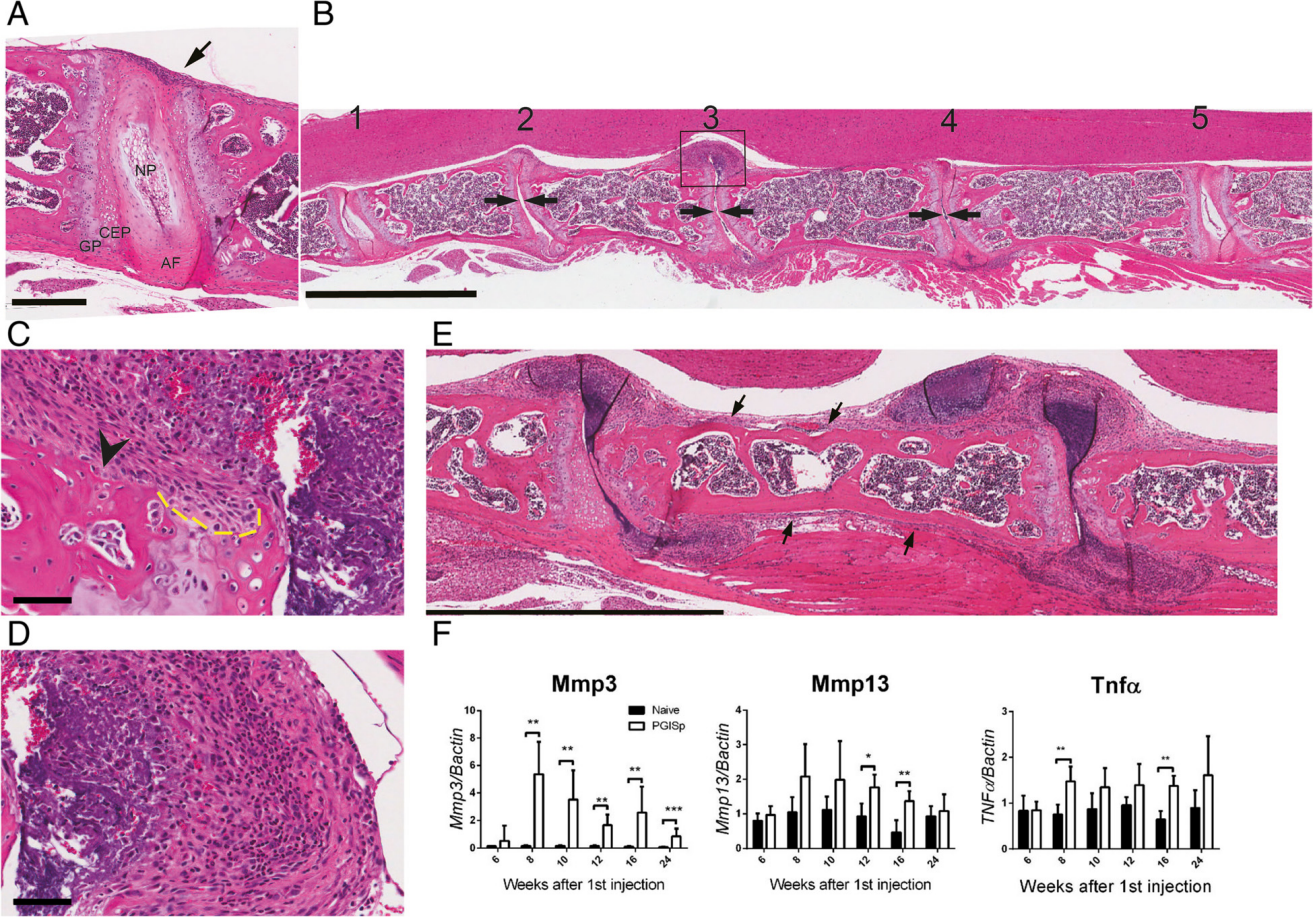
Progression of spinal inflammation in PGISp mice. **a** Representative H&E stain of mild inflammation in an 8-week time point mouse. Inflammatory cells accumulate at the periphery of the disc (*arrow*), but the rest of the intervertebral disc appears normal. *AF* annulus fibrosus, *CEP* cartilaginous end plate, *GP* cartilaginous growth plate, *NP* nucleus pulposus. Scale bar = 300 μm. **b** Variable disease penetrance in an 8-week PGISp mouse: (1) and (5) unaffected intervertebral discs, (2) disc destruction combined with early chondrocyte formation, (3) severe inflammation and disc destruction, and (4) moderate inflammation with incomplete disc destruction. Scale bar = 2 mm. **c** Magnification of the boxed area in (b) shows bone erosion (*arrowhead*) and cartilage damage. The yellow dashed line indicates the eroded cartilage surface. Scale bar = 60 μm. **d** Magnification of the boxed area in (b) demonstrating large numbers of mononuclear cells presenting in the affected joints. Scale bar = 60 μm. **e** Inflammation expansion along the longitudinal ligament (*arrows*). **f** mRNA expression of *Mmp3*, *Mmp13* and *Tnf* in whole spine was analysed by qPCR and normalized to *β-actin*. Black bars represent naïve and open bars represent PGISp mice. Expression levels are presented as mean ± standard deviation. ***P < 0.0001, **P < 0.001, *P < 0.01 compared with naïve at the same time point, Mann-Whitney test. *MMP* matrix metalloproteinase, *TNFα* tumour necrosis factor-alpha

Detailed histological assessment across the time course exemplified the heterogeneity of disease progression between different animals and even between individual vertebral joints, similar to that reported previously in this model [7, 8] and in AS [10]. At the 6-week time point, the earliest detected feature of inflammation was accumulation of inflammatory cells at the periphery of the IVD adjacent to the annulus fibrosus (AF) (Fig. 2a). At 8 weeks after priming, while all mice were affected, within any given PGISp mouse, both affected (Fig. 2b, joints 2 to 4) and unaffected (Fig. 2b, joints 1 and 5) vertebral joints were observed and there was a broad range of inflammation severity within a single spine (Fig. 2b, joints 2–4). Inflammatory pannus invading into the disc space was clearly associated with IVD destruction, resulting in joint space narrowing (Fig. 2b, space between arrows), and was often associated with bone erosion (Fig. 2c, arrowhead). Early hyaline cartilage damage, including eroded surfaces defined by excavated and empty chondrocyte lacunae, was confined to areas exposed to inflammatory infiltrate (Fig. 2c, yellow dash line represented eroded cartilage surface), similar to previous observation in vertebrae and sacroiliac joints in PGISp mice [7, 8].

Severe inflammation was characterised by marked infiltration of the AF by inflammatory cells, including polymorphonuclear cells, vascular structures and increased density of mixed morphology mesenchymal cells (Fig. 2c and d). In severely inflamed joints, periosteal expansion and inflammatory cell invasion frequently extended along the cortical surface of the vertebral body, possibly following the spinal ligaments, suggesting an entheseal or periosteal pathology or both (Fig. 2e, arrows). Overall the histopathology features suggest that the inflammatory process drives the IVD destruction, focal cartilage damage and focal bone erosion, particularly at the vertebral corners, all of which are reported features of AS axial pathology.

TNFα and matrix metalloproteinase-3 (MMP-3) and MMP-13 have been implicated in AS inflammatory processes and have been correlated with disease activity [11–13]. Therefore, mRNA expression of these genes in the whole spine was measured by qPCR (Fig. 2f). The basal level of *Mmp3* in unaffected mice was very low across the time course (Fig. 2f, black bars). PG immunisation induced a dramatic elevation of *Mmp3* at 8 weeks and expression levels of this transcript remained significantly higher in PGISp mice when compared with naive mice at all later time points (Fig. 2f). *Mmp13* was significantly elevated at weeks 12 and 16 in PGISp spines. *Tnf* expression was significantly increased in PGISp mice at 8 and 16 weeks (Fig. 2f). These data suggest that the inflammation-driven tissue remodelling molecular cascades are established by 8–10 weeks post-immunisation and decrease in parallel with inflammation during the later disease stages.

### Disease-associated anabolic tissue formation

**Fig. 3:**
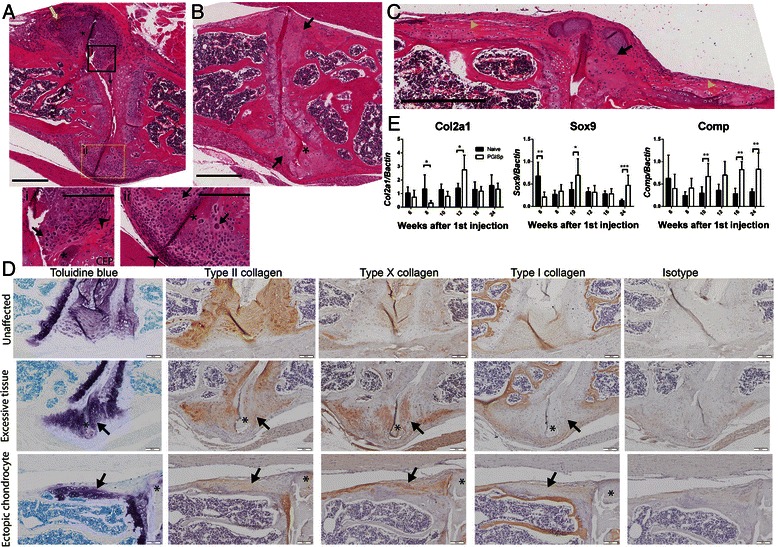
Excessive tissue and ectopic chondrocyte formation are key features of advanced disease. **a** A 12-week mouse IVD demonstrates both early (i) and intermediate (ii) stages of excessive tissue formation using H&E stain. Scale bar = 300 μm. Inflammatory infiltrate (*yellow arrow*) surrounds the affected joint, while fibroblast-like cells (*arrowheads*) and chondrocyte-like cells (*arrows*) are located adjacently to the residual disc (*asterisks*). Scale bar = 200 μm in (i) and (ii). *CEP* cartilaginous end plate. **b** Excessive tissue at the periphery of the joint is greatly increased in more advanced disease (*arrows*). Representative image from a 24-week mouse. Scale bar = 400 μm. **c** In addition to excess tissue (*black arrow*), columnar chondrocytes expand ectopically along the cortical bone between affected joints (*yellow arrowheads*). Representative image from a 24-week mouse. Scale bar = 300 μm. **d** Representative images taken from 24-week mice show an unaffected IVD and affected joints with excessive or ectopic chondrocyte formation. Cartilaginous tissue (*arrows*) is positive for toluidine blue (proteoglycan content) and type II collagen. Type X collagen stains for hypertrophic chondrocytes while type I collagen delineates mature bone. Scale bar = 100 μm. **e** Spinal gene expression profiles of cartilage markers *Col2a1*, *Comp* and *Sox9* were analysed by qPCR reaction and normalized to β-actin. Black bars represent naïve and open bars represent PGISp mice. Expression levels are presented as mean ± standard deviation. ***P < 0.0001, **P < 0.001, *P < 0.01 compared with naïve at the same time point, Mann-Whitney test. *Col2a1* type II collagen, α 1, *COMP* cartilage oligomeric matrix protein, *Sox9* sex determining region Y- box 9

Expansion of mesenchymal cells, predominantly fibroblast-like (Fig. 3a i and ii, arrowheads) and chondrocyte-like (Fig. 3a i and ii, arrows), was noted in the bulging residual AF of affected vertebral joints and extending along the vertebral periosteal surface (Fig. 3a). The chondrocyte-like cell phenotype included rounded or polygonal morphology within a dense predominantly basophilic extracellular matrix (ECM) (Fig. 3a i and ii, arrows). In advanced disease, ectopic cartilaginous tissue increased as the inflammatory cell infiltrate and density of fibroblast-like mesenchymal cells declined (Fig. 3b, arrows). Ectopic chondrogenesis/chondrophyte formation occurred only following disc destruction, and residual necrotic disk tissue was frequently evident (Fig. 3a, asterisks). Ectopic cartilage-like tissue containing embedded chondrocytes in columnar formation parallel to the vertebral periosteal cortical surface was also observed in some mice and was possibly associated with longitudinal ligament attachment points (Fig. 3c, yellow arrowheads). Interestingly, the cortical bone underlying the columnar cartilage-like tissues had evidence of erosive damage, including pitting of the periosteal surface (Fig. 2c). Inflammation was noted in similar regions of other mice (Fig. 2e, arrows), as were cells with chondroblast-like morphology embedded within dense ECM (Figs. 3aii and b). These features were not observed concomitantly on any given vertebral body, suggesting that while they occurred at a similar anatomical location, they were temporally staggered processes, although owing to cross-sectional experimental design, these observations are correlative.

The composition of the ectopic ECM present at sites of PGISp excess tissue formation was determined by assessing distribution of different types of collagen by immunohistochemistry (Fig. 3d). In unaffected vertebral joints (Fig. 3d, top row), the AF and endplate hyaline cartilage had high PG content (toluidine blue-positive) and predominantly contained type II collagen. Type I collagen was weakly present in AF but was a major component of the vertebral bone. In affected joints (Fig. 3d, middle and bottom row), the majority of the excessive tissue (Fig. 3d, arrows) had a high PG content and contained both type II and type X collagens, verifying pathologic chondroproliferation. Expression of type X collagen suggested that some chondrocytes were hypertrophic. Consistent with previous findings, weak collagen type I staining seen at the periphery of excessive tissue areas, potentially indicated progression towards formation of bone [8].

Spinal mRNA expression of cartilage matrix components and cartilage-forming regulatory proteins fluctuated during disease progression (Fig. 3e). Expression of cartilage components *col2a1* and *Sox9* was decreased at 8 and 6 weeks post-priming, respectively. From 10 weeks onwards, as inflammation peaked and excessive tissue formation increased, cartilage oligomeric matrix protein (*Comp*) was significantly elevated. *Col2a1* was upregulated at week 12 whereas *Sox9* increased at weeks 10 and 24. This trend suggests cartilaginous matrix expression was increased in PGISp mice after the peak of inflammation.

**Fig. 4:**
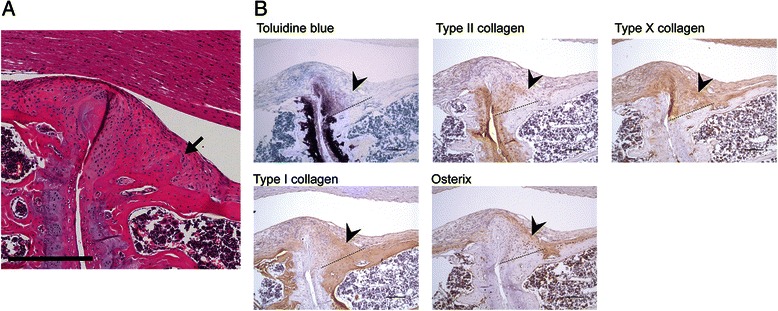
New bone and osteophyte formation. **a** Representative image of an osteophyte (*arrow*) in the affected joint of a 12-week proteoglycan-induced spondylitis mouse. Scale bar = 300 μm. **b** Recently formed bone tissue (*arrowheads*) is characterised by a transition from proteoglycan and type II collagen-enriched matrix to type X and type I collagen-positive matrix. Osterix-positive osteoblasts are embedded in type I collagen-positive bone matrix. The dashed line represents the boundary between the original cortical bone and the excessive matrix above, showing a reduction of cartilaginous matrix, toluidine blue staining and type II collagen accompanied with an increase of type I collagen and osterix-expressing osteoblasts. *Arrowheads* indicate cartilage tissue that is strongly positive for type II collagen but weakly for type I collagen. Scale bar = 100 μm.

Mature osteophyte formation, defined as aberrant bone formation adjacent to cortical bone (Fig. 4a), was apparent. Osteophytes showed a variable collagen distribution with different areas containing collagen I-, II- or X-positive and osterix-positive cells (Fig. 4b). None of the PGISp mice at weeks 6 and 8 developed osteophytes, but 28.6 % of mice at week 10 had osteophytes, and the percentage increased to 71.4 %, 66.7 % and 75 % at weeks 12, 16 and 24, respectively. A noteworthy observation is that pathological tissue formation was observed only with vertebral joints that exhibited IVD destruction.

### Modelling of disease

**Fig. 5:**
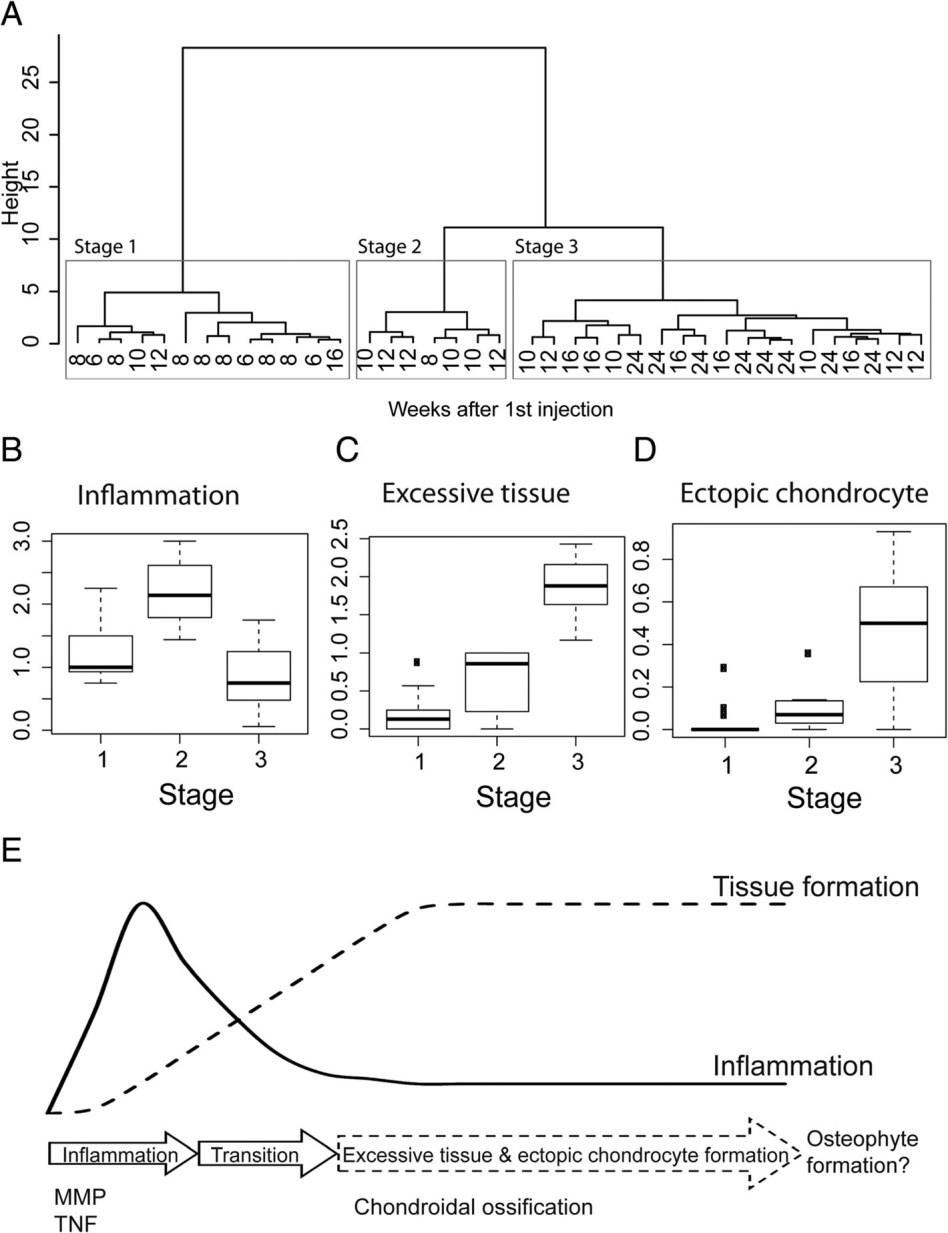
Early, intermediate and late phases of disease progression are delineated by inflammation, excessive tissue and ectopic chondrocyte formation. **a** Unsupervised clustering differentiates the model into three stages corresponding with disease duration. The x-axis depicts the time points of the mice. The left box (stage 1), middle box (stage 2) and right box (stage 3) represent early, intermediate and late stages of disease development, respectively. Scores for (**b**) inflammation, (**c**) excessive tissue formation and (**d**) ectopic chondrocyte formation are distinct between these three groups. **e** Axial disease was initially characterised by transient inflammation that included vertebral joint infiltration by monocular cells, activation of tissue remodelling (matrix metalloproteinases, or MMPs) and pro-arthritic inflammatory pathways (tumour necrosis factor, or TNF). Vertebral joint inflammation culminated in destructive changes, including destruction of the intervertebral disc, the latter of which was irreparable and would have considerable impact on joint biomechanics. In advanced disease, inflammation is decreased; however, excessive tissue and ectopic chondrocyte formation driven by chondroidal ossification was the predominant feature and occurred only in joints in which the IVD had been severely compromised

To unbiasedly model the interdependence of the different scoring criteria irrespectively of time post-PG priming, we developed an algorithm to define different disease stages. This algorithm used an unsupervised clustering approach based purely on average affected vertebral joint scores across all the criteria measured. This clustering defined three disease stages which interestingly corresponded broadly to the time point post-priming—stage 1: early (mainly week 6 to 8, Fig. 5a, left box); stage 2: intermediate (mainly weeks 10 to 12, Fig. 5a, middle box); and stage 3: advanced (mainly weeks 16–24, Fig. 5a, right box). Stage 3 could also be divided into two subgroups with a very late cluster consisting of almost entirely 24-week mice. We further dissected the data to identify the scoring metrics that best delineated each stage. The scores for inflammation (Fig. 5b), excessive tissue formation (Fig. 5c) and ectopic chondrocyte formation (Fig. 5d) strongly differentiated between these three groups. The early stage showed mild to moderate inflammation, low excessive tissue and low ectopic chondrocyte formation. The intermediate stage displayed severe inflammation and moderate excessive tissue and ectopic chondrocyte formation. The advanced stage showed decreased inflammation but pronounced excess tissue and ectopic chondrocyte formation.

## Discussion

Inflammation and syndesmophyte formation are two defining hallmarks in AS. However, the relationships between these features, as well as the underlying controlling factors, are poorly understood. Key questions that remain to be answered are whether the enthesis is the site of disease initiation, whether inflammation is necessary for radiographic disease progression, and what is the nature of the pathological osseous tissue laid down. By detailed analysis of disease progression in the PGISp mouse model of AS, we have provided insights into these questions. Specifically we have demonstrated that osteoproliferation is seen only at sites where inflammation previously occurred, strongly suggesting that inflammation and IVD destruction are required for disease progression to bone formation.

The detailed histology analysis in the present study revealed that the inflammatory invasion started at the periphery of the AF. Association of inflammation with the AF has been previously noted in this model but was not identified as the inflammation initiating point within spine [7, 8]. Spinal enthesitis presenting as pannus formation outside of the AF was also reported in HLA-B27 transgenic rats [14] and curdlan-treated SKG mice [15]. We also showed that inflammatory infiltrate and ectopic chondroproliferation expanded along the adjacent vertebrae cortical bone surfaces. Although we do not show definitive proof of entheses in these regions, it is well established that the longitudinal spinal ligaments have frequent attachment points in this area [16, 17].

The limited effects of anti-TNF therapies on radiographic progression raised the question as to whether inflammation is required for osteoproliferation in AS. Our detailed histological examination suggests inflammation, excessive tissue formation and ectopic chondrocyte formation typify early, intermediate and late disease stages, respectively. We demonstrated that early mesenchymal expansion initiated before resolution of inflammation. Although averaging of histological scores suggests overlap between the disease stages, examination of individual vertebrae showed that severe inflammation was never found simultaneously with severe excessive tissue formation within the same joint, supporting sequential rather than parallel progression of these disease features. Therefore, our model predicts that inflammatory lesions are likely to have decreased, if not resolved, in the later disease stages typified by syndesmophyte formation. This prediction is supported by MRI studies in AS patients that have shown that (1) new syndesmophytes are seen more frequently in vertebral corners with resolved inflammation [18] and (2) 68 % of syndesmophytes developed from vertebral corners without active inflammation during the 2-year observation period [19]. However, some consideration needs to be given to the sensitivity limits of MRI for detecting mild inflammation [20].

Excessive tissue formation occurred only at vertebral joints in which the IVD itself had been destroyed, implying that disease initiates with inflammation which leads to disc destruction. As inflammation decreases, excessive tissue formation commences with ectopic chondrocyte formation occurring late. Such a progression concurs with the “TNF-brake hypothesis” proposed by Maksymowych *et al*. [18], who suggest that a high level of inflammatory TNF actually inhibits osteoproliferation and this inhibition is eased as TNF levels drop with resolution of inflammation. This also matches data from rheumatoid arthritis models in which osteoblast function is inhibited in the presence of inflammation [21] and induced after inflammation resolution [22]. However, we also see elevated TNF expression in the spines of the PGISp mice from inflammation throughout the excess tissue production phases, suggesting that TNF may play a role in the osteoproliferation as well as inflammation. Alternatively, these elevated levels might also reflect the disease heterogeneity within individual vertebrae and local TNF levels may result in site-specific impacts on osteoblast regulation.

Enthesitis and osteoproliferation have also been reported in DBA/1 mice [23], HLA-B27/hβ2m transgenic rats [24] and curdlan-treated SKG mice [15]. However, the relationship between the processes is not well defined. In the DBA/1 mouse model of ankylosing enthesitis, chondrocyte proliferation was the leading cause of ankle ankylosis [23, 25]. The entheseal proliferation and inflammatory infiltrate were minor in this model, and treatment with glucocorticoids [26], but not etanercept [27], did ameliorate inflammation but had no effect on the ankylosis. In a longitudinal study in HLA-B27/hβ2m transgenic rats, osteoproliferation was found external to the joint space separate to inflammatory infiltrates [24] but was present simultaneously, suggesting distinct but parallel processes. Interestingly, although blocking TNF signalling before or after onset prevented or reduced arthritis, respectively [28], chondroproliferation and its activation of relevant bone morphogenetic protein signalling were prevented only by early treatment. In the SKG mouse model, bone formation and erosion were shown to be adjacent to inflammation, although progressive association of these different features is unclear as a detailed longitudinal study was not undertaken [15]. So although evidence from other animal models is ambiguous, our extensive analysis in the PGISp model clearly supports a direct progression from inflammation to cartilage-bone formation.

Direct connection of the ectopic chondrocytes and cartilage to the vertebral end plate or growth plate cartilage was not a consistent histological feature in our model, indicating that these ectopic tissues were not due to expansion of existing cartilage structures. The AF [29], periosteum [30] and ligament [16] all contain potential chondrocyte progenitors. Therefore, it is possible the chondrogenesis is initiated using progenitors located within these disease-affected tissues without direct involvement of pre-existing cartilage. Several features of the excessive tissue in the advanced disease, such as cartilage formation and chondrocyte hypertrophy, resembled endochondral ossification. Hypertrophic chondrocytes have been shown to be able to transform directly into osteoblasts and osteocytes [31]. Bleil *et al*. recently demonstrated that zygapophyseal joint ankylosis was caused by direct transformation from cartilage to bone without chondrocyte hypertrophy [32]. The direct transformation/ossification from chondrocytes represents chondroidal ossification [2, 32] and might be the primary mechanism of excessive tissue formation in the PGISp model. Examples of mature syndesmophytes were rare in this study but more evident in later disease stages. This finding suggests that the transformation of cartilage to bone in PGISp mice is a slow process modelling the prolonged window between disease onset and radiographic change in patients with AS [33].

Our results suggest that if inflammation is not treated early enough to prevent irreparable structural damage, further osteoproliferation at that location will progress even if the inflammatory process is resolved/attenuated. The benefits of early intervention regarding both spondylitis and radiographic progression have been demonstrated with different treatments. After treatment with infliximab for 16 weeks, 61 % of patients with short disease duration (13.4 months) had 40 % improvement from baseline [34] whereas only 47 % of patients with long disease duration (7.7 years) reached the same criteria after 24 weeks of treatment [35]. Delaying TNF intervention therapy for 10 years was related to faster radiographic progression compared with those who started treatment within 10 years of disease onset [36]. The benefit of early intervention was also seen using NSAIDs. AS patients with short disease duration (<5 years) had less radiographic progression [37] compared with those with long disease duration (11.9 years) [38] following chronic high-dose NSAID treatment. These studies suggest that anti-inflammatory therapies exert better clinical outcomes in patients with early AS/spondyloarthritis compared with those whose disease is established.

## Conclusions

In summary, our study indicates inflammation leads to IVD destruction in the PGISp mouse AS model. The latter is a prerequisite for induction of excessive tissue formation, and chondroidal ossification rather than endochondral or intramembranous ossification appears to be the most likely mechanism. Extrapolation of these findings to human disease suggests that early intervention with potent anti-inflammatory therapeutic regimens may prevent inflammation-induced destructive changes and consequently reduce the frequency of osteoproliferative events and joint fusion.

## Additional file

Additional file 1: Table S1. Antibodies for immunohistochemistry. **Table S2.** Primers for real-time PCR. (DOC 36 kb)

## Abbreviations

AF: Annulus fibrosus
AS: Ankylosing spondylitis
Col2a1: Type II collagen, α 1
ECM: Extracellular matrix
EDTA: Ethylenediaminetetraacetic acid disodium salt dehydrate
H&E: Haematoxylin and eosin
HLA: Human leukocyte antigen
IVD: Intervertebral disc
MMP: Matrix metalloproteinase
MRI: Magnetic resonance imaging
NSAID: Non-steroidal anti-inflammatory drug
PCR: Polymerase chain reaction
PG: Proteoglycan
PGISp: Proteoglycan-induced spondylitis
qPCR: Quantitative real-time reverse transcription polymerase chain reaction
RT: Room temperature
Sox9: Sex determining region Y- box 9
TBS: Tris-buffered saline
TNF: Tumour necrosis factor

## References

[1] Benjamin M, Toumi H, Suzuki D, Hayashi K, McGonagle D (2009). Evidence for a distinctive pattern of bone formation in enthesophytes. Ann Rheum Dis.

[2] Francois RJ, Gardner DL, Degrave EJ, Bywaters EG (2000). Histopathologic evidence that sacroiliitis in ankylosing spondylitis is not merely enthesitis. Arthritis Rheum.

[3] Appel H, Maier R, Wu P, Scheer R, Hempfing A, Kayser R (2011). Analysis of IL-17+ cells in facet joints of patients with spondyloarthritis suggests that the innate immune pathway might be of greater relevance than the Th17-mediated adaptive immune response. Arthritis Res Ther.

[4] Appel H, Kuhne M, Spiekermann S, Ebhardt H, Grozdanovic Z, Kohler D (2006). Immunohistologic analysis of zygapophyseal joints in patients with ankylosing spondylitis. Arthritis Rheum.

[5] Appel H, Kuhne M, Spiekermann S, Kohler D, Zacher J, Stein H (2006). Immunohistochemical analysis of hip arthritis in ankylosing spondylitis: evaluation of the bone-cartilage interface and subchondral bone marrow. Arthritis Rheum.

[6] Appel H, Maier R, Bleil J, Hempfing A, Loddenkemper C, Schlichting U (2013). In situ analysis of interleukin-23- and interleukin-12-positive cells in the spine of patients with ankylosing spondylitis. Arthritis Rheum.

[7] Bárdos T, Szabó Z, Czipri M, Vermes C, Tunyogi-Csapó M, Urban RM (2005). A longitudinal study on an autoimmune murine model of ankylosing spondylitis. Ann Rheum Dis.

[8] Haynes KR, Pettit AR, Duan R, Tseng HW, Glant TT, Brown MA (2012). Excessive bone formation in a mouse model of ankylosing spondylitis is associated with decreases in Wnt pathway inhibitors. Arthritis Res Ther.

[9] Finnegan A, Grusby MJ, Kaplan CD, O’Neill SK, Eibel H, Koreny T (2002). IL-4 and IL-12 regulate proteoglycan-induced arthritis through Stat-dependent mechanisms. J Immunol.

[10] Calin A, Elswood J (1988). The relationship between pelvic, spinal and hip involvement in ankylosing spondylitis--one disease process or several?. Br J Rheumatol.

[11] Yang C, Gu J, Rihl M, Baeten D, Huang F, Zhao M (2004). Serum levels of matrix metalloproteinase 3 and macrophage colony-stimulating factor 1 correlate with disease activity in ankylosing spondylitis. Arthritis Rheum.

[12] Soliman E, Labib W, el-Tantawi G, Hamimy A, Alhadidy A, Aldawoudy A (2012). Role of matrix metalloproteinase-3 (MMP-3) and magnetic resonance imaging of sacroiliitis in assessing disease activity in ankylosing spondylitis. Rheumatol Int.

[13] Arends S, van der Veer E, Groen H, Houtman PM, Jansen TL, Leijsma MK (2011). Serum MMP-3 level as a biomarker for monitoring and predicting response to etanercept treatment in ankylosing spondylitis. J Rheumatol.

[14] Hammer RE, Maika SD, Richardson JA, Tang JP, Taurog JD (1990). Spontaneous inflammatory disease in transgenic rats expressing HLA-B27 and human beta 2 m: an animal model of HLA-B27-associated human disorders. Cell.

[15] Ruutu M, Thomas G, Steck R, Degli-Esposti MA, Zinkernagel MS, Alexander K (2012). beta-glucan triggers spondylarthritis and Crohn’s disease-like ileitis in SKG mice. Arthritis Rheum.

[16] Cai HX, Yayama T, Uchida K, Nakajima H, Sugita D, Guerrero AR (2012). Cyclic tensile strain facilitates the ossification of ligamentum flavum through beta-catenin signaling pathway: in vitro analysis. Spine (Phila Pa 1976).

[17] Loughenbury PR, Wadhwani S, Soames RW (2006). The posterior longitudinal ligament and peridural (epidural) membrane. Clin Anat.

[18] Maksymowych WP, Chiowchanwisawakit P, Clare T, Pedersen SJ, Ostergaard M, Lambert RG (2009). Inflammatory lesions of the spine on magnetic resonance imaging predict the development of new syndesmophytes in ankylosing spondylitis: evidence of a relationship between inflammation and new bone formation. Arthritis Rheum.

[19] van der Heijde D, Machado P, Braun J, Hermann KG, Baraliakos X, Hsu B (2012). MRI inflammation at the vertebral unit only marginally predicts new syndesmophyte formation: a multilevel analysis in patients with ankylosing spondylitis. Ann Rheum Dis.

[20] Appel H, Loddenkemper C, Grozdanovic Z, Ebhardt H, Dreimann M, Hempfing A (2006). Correlation of histopathological findings and magnetic resonance imaging in the spine of patients with ankylosing spondylitis. Arthritis Res Ther.

[21] Walsh NC, Reinwald S, Manning CA, Condon KW, Iwata K, Burr DB (2009). Osteoblast function is compromised at sites of focal bone erosion in inflammatory arthritis. J Bone Miner Res.

[22] Matzelle MM, Gallant MA, Condon KW, Walsh NC, Manning CA, Stein GS (2012). Resolution of inflammation induces osteoblast function and regulates the Wnt signaling pathway. Arthritis Rheum.

[23] Corthay A, Hansson AS, Holmdahl R (2000). T lymphocytes are not required for the spontaneous development of entheseal ossification leading to marginal ankylosis in the DBA/1 mouse. Arthritis Rheum.

[24] van Duivenvoorde LM, Dorris ML, Satumtira N, van Tok MN, Redlich K, Tak PP (2012). Relationship between inflammation, bone destruction, and osteoproliferation in the HLA-B27/human beta(2) -microglobulin-transgenic rat model of spondylarthritis. Arthritis Rheum.

[25] Lories RJ, Matthys P, de Vlam K, Derese I, Luyten FP (2004). Ankylosing enthesitis, dactylitis, and onychoperiostitis in male DBA/1 mice: a model of psoriatic arthritis. Ann Rheum Dis.

[26] Braem K, Deroose CM, Luyten FP, Lories RJ (2012). Inhibition of inflammation but not ankylosis by glucocorticoids in mice: further evidence for the entheseal stress hypothesis. Arthritis Res Ther.

[27] Lories RJ, Derese I, de Bari C, Luyten FP (2007). Evidence for uncoupling of inflammation and joint remodeling in a mouse model of spondylarthritis. Arthritis Rheum.

[28] Milia AF, Ibba-Manneschi L, Manetti M, Benelli G, Generini S, Messerini L (2011). Evidence for the prevention of enthesitis in HLA-B27/hbeta(2)m transgenic rats treated with a monoclonal antibody against TNF-alpha. J Cell Mol Med.

[29] Nosikova Y, Santerre JP, Grynpas MD, Kandel RA (2013). Annulus fibrosus cells can induce mineralization: an in vitro study. Spine J.

[30] Ito Y, Fitzsimmons JS, Sanyal A, Mello MA, Mukherjee N, O’Driscoll SW (2001). Localization of chondrocyte precursors in periosteum. Osteoarthritis Cartilage.

[31] Yang L, Tsang KY, Tang HC, Chan D, Cheah KS (2014). Hypertrophic chondrocytes can become osteoblasts and osteocytes in endochondral bone formation. Proc Natl Acad Sci U S A.

[32] Bleil J, Maier R, Hempfing A, Schlichting U, Appel H, Sieper J (2014). Histomorphologic and histomorphometric characteristics of zygapophyseal joint remodeling in ankylosing spondylitis. Arthritis Rheumatol.

[33] van Tubergen A, Weber U (2012). Diagnosis and classification in spondyloarthritis: identifying a chameleon. Nat Rev Rheumatol.

[34] Barkham N, Keen HI, Coates LC, O’Connor P, Hensor E, Fraser AD (2009). Clinical and imaging efficacy of infliximab in HLA-B27-Positive patients with magnetic resonance imaging-determined early sacroiliitis. Arthritis Rheum.

[35] van der Heijde D, Dijkmans B, Geusens P, Sieper J, DeWoody K, Williamson P (2005). Efficacy and safety of infliximab in patients with ankylosing spondylitis: results of a randomized, placebo-controlled trial (ASSERT). Arthritis Rheum.

[36] Haroon N, Inman RD, Learch TJ, Weisman MH, Lee M, Rahbar MH (2013). The impact of tumor necrosis factor alpha inhibitors on radiographic progression in ankylosing spondylitis. Arthritis Rheum.

[37] Poddubnyy D, Haibel H, Listing J, Marker-Hermann E, Zeidler H, Braun J (2012). Baseline radiographic damage, elevated acute-phase reactant levels, and cigarette smoking status predict spinal radiographic progression in early axial spondylarthritis. Arthritis Rheum.

[38] Wanders A, Heijde D, Landewe R, Behier JM, Calin A, Olivieri I (2005). Nonsteroidal antiinflammatory drugs reduce radiographic progression in patients with ankylosing spondylitis: a randomized clinical trial. Arthritis Rheum.

