# Supplementary material for: Inflammation-driven bone formation in a mouse model of ankylosing spondylitis: sequential not parallel processes
Source: Arthritis Res Ther. 2016 Jan 29;18:35. doi: 10.1186/s13075-015-0805-0 (PMC4734853; doi:10.1186/s13075-015-0805-0)
Supplement: Additional file 1: Table S1. — Antibodies for immunohistochemistry. Table S2. Primers for real-time PCR. (DOC 36 kb) [file 13075_2015_805_MOESM1_ESM.doc]

Table S1: Antibodies for immunohistochemistry

| Antibody | **Concentration (dilution)** | Titre | Cetology number | Company |
| --- | --- | --- | --- | --- |
| Rabbit anti-Type I collagen | 0.5 μg/ml (1:2000) | 1:2000 | C7510-13 | USBioLogical, Swanpscott, MA |
| Rabbit anti-Type II collagen | 0.5 μg/ml (1:2000) | 1:2000 | Ab34712 | Abcam, Cambridge, UK |
| Rabbit anti-Type X collagen | 1:8000 | 1:8000 | LSL-LB-0092 | Cosmo bioco, Tokyo, Japan |
| Rabbit anti-Osterix | 0.2 μg/ml (1:4000) | 1:4000 | Ab22552 | Abcam, Cambridge, UK |
| Rabbit IgG | Same concentration as antibody | Same concentration as antibody | Sc-2027 | Santa Cruz Biotechnology, Santa Cruz, CA |
| Rabbit serum | 1:8000 | 1:8000 | R9133 | Sigma-Aldrich |

Table S2: Primers for real-time PCR

| Gene | Accession Number | Forward Primer (5’-3’) | Reverse Primer (3’-5’) |
| --- | --- | --- | --- |
| β-actin | NM_007393 | GATTACTGCTCTGGCTCCTAG | GACTCATCGTACTCCTGCTTG |
| Col2a1 | NM_031163 | GCAGAGATGGAGAACCTGGTA | AGCCTTCTCGTCATACCCT |
| Comp | NM_016685 | GTCCAAGAAGAATGACGATCAGA | ACAGTTGTCAGCTACATTTCGT |
| Mmp3 | NM_010809 | GATGAACGATGGACAGAGGATG | TGTGGAGGACTTGTAGACTGG |
| Mmp13 | NM_008607 | GCCATTTCATGCTTCCTGATG | AGACTGGTAATGGCATCAAGG |
| Sox9 | NM_011448 | CGACCCATGAACGCCTT | GTCTCTTCTCGCTCTCGTTC |
| Tnfα | NM_013693 | AGACCCTCACACTCAGATCA | TCTTTGAGATCCATGCCGTTG |

Cartilage oligomeric matrix protein (Comp); Matrix metalloproteinase (MMP); Tumor necrosis factor alpha (Tnfα); Type II collagen, α 1 (col2a1); Sex determining region Y- box 9 (Sox9)
